# Cytokine concentrations in pediatric primary mediastinal large B-cell lymphoma correlate with disease extent and outcome

**DOI:** 10.1182/bloodadvances.2026021463

**Published:** 2026-09-12

**Authors:** Malte Nipper, Christine Damm-Welk, Will Shepheard, Ilske Oschlies, Wolfram Klapper, Birgit Burkhardt, Wilhelm Woessmann, Fabian Knörr

**Affiliations:** 1 Pediatric Hematology and Oncology and NHL-BFM Study Center, University Medical Center Hamburg-Eppendorf, Hamburg, Germany; 2 Research Institute Children's Cancer Center Hamburg, Hamburg, Germany; 3 Section for Hematopathology and Lymph Node Registry, Department of Pathology, University Hospital Schleswig-Holstein, Kiel, Germany; 4 Pediatric Hematology and Oncology, University Hospital Muenster, Muenster, Germany

## Abstract

Primary mediastinal large B-cell lymphoma (PMBCL) predominantly affects female adolescents and young adults. It displays an immune-privileged phenotype and demonstrates the involvement of key cytokine signaling pathways, including increased expression of thymus- and activation-regulated chemokine (TARC/CC chemokine ligand 17 [CCL17]). Currently, there is a lack of established markers for stratification. We studied plasma concentrations of 24 cytokines at diagnosis in a large population–based pediatric cohort of 62 patients with PMBCL. Compared with a group of age-matched patients with other types of lymphomas in long-term remission and healthy individuals (n = 26), patients with PMBCL showed elevated concentrations of CCL4, CCL17, interleukin-6 (IL-6), CXC chemokine ligand 8 (CXCL8), CXCL9, CXCL10, and CXCL11. The median CCL17 level was 1695 pg/mL (interquartile range [IQR], 642-3142) in patients with PMBCL compared with 81 pg/mL (IQR, 41-190) in controls (*P* < .0001). The concentrations of CCL17, CXCL9, and CXCL10 significantly correlated with mediastinal tumor volume (MTV) and lactate dehydrogenase (LDH) activity but were not associated with event-free survival (EFS). The concentrations of IL-17F and IL-22 were inversely correlated with LDH activity and MTV. Elevated IL-6, IL-10, IL-17A, and IL-22 levels were significantly correlated with inferior EFS in a subgroup of 50 patients uniformly treated with dose-adjusted (DA)-EPOCH-R (etoposide, prednisone, oncovin [vincristine], cyclophosphamide, doxorubicin, and rituximab). Although based on a limited number of events, IL-6 level showed the strongest association with outcome (hazard ratio, 6.8; 95% confidence interval, 1.5-30.9). In summary, pretreatment cytokine levels in patients with PMBCL reveal distinct patterns associated with tumor burden (CCL17, CXCL9, and CXCL10) and outcome (IL-6, IL-10, IL-17A, and IL-22).

## Key Points

• Concentrations of CCL17, CXCL9, and CXCL10 at diagnosis correlated with disease extent in pediatric patients with PMBCL.
• IL-6, IL-10, IL-17A, and IL-22 levels were associated with inferior EFS in patients treated with dose-adjusted EPOCH with rituximab.

## Visual Abstract

**Figure:**
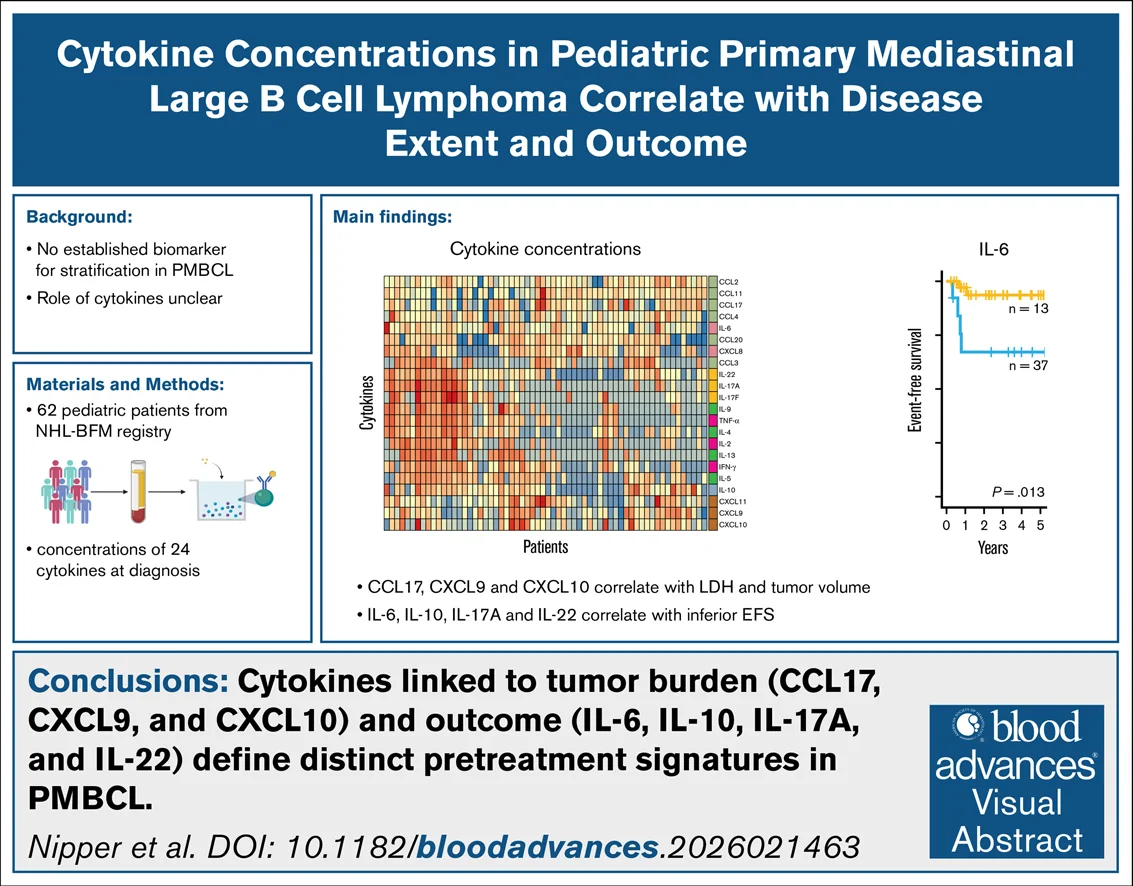

## Introduction

Primary mediastinal large B-cell lymphoma (PMBCL) predominantly affects female adolescents and young adults. Patients frequently present with a bulky mediastinal mass and compressive symptoms, often requiring urgent intervention. PMBCL is characterized by the constitutive activation of the NF-κB and Janus kinase (JAK)/signal transducers and activator of transcription (STAT) cytokine signaling pathways,^1^^,^^2^ as well as the immune-privileged phenotype resulting, among others, from the amplification of PD-L1/PD-L2.^3^

Cytokines are potential biomarkers in infectious, inflammatory, and malignant conditions, including Hodgkin lymphoma,^4^ diffuse large B-cell lymphoma (DLBCL),^5^ and ALK-positive anaplastic large cell lymphoma (ALCL).^6^ Cytokine-mediated B symptoms are frequently observed in patients with PMBCL.^7^ In PMBCL, the upregulation of the NF-κB pathway leads to the activation of antiapoptotic and prosurvival genes, the upregulation of PD-L1/PD-L2 expression, and a chemokine response of thymus- and activation-regulated chemokine (TARC/CC chemokine ligand 17 [CCL17]).^8^^,^^9^ The constitutive activation of the JAK/STAT pathways, driven by an amplification of JAK2 and mutations in regulatory genes such as SOCS1,^10^ results in increased cellular proliferation and cytokine signaling, also including CCL17.^9^^,^^11^^,^^12^ CCL17 is typically highly elevated in patients with Hodgkin lymphoma,^13^ a disease sharing biological characteristics with PMBCL, and has also been evaluated as a high-risk marker in lymphoblastic lymphoma.^14^ However, systemic data from cohorts of patients with PMBCL have been lacking.

Dose-intensive chemoimmunotherapy regimens have improved response rates^15^ and treatment by dose-adjusted (DA)-EPOCH-R (etoposide, prednisone, cyclophosphamide, doxorubicin, and rituximab) results in event-free survival (EFS) rates of 80% to 85% and overall survival of ∼90% to 95% in studies of adult and pediatric cohorts.^16^, ^17^, ^18^ Given the intensity of current treatments with their risks for late effects and the relapse rate, there is an urgent need for prognostic parameters allowing for improved patient stratification for both deescalation studies and intensification for high-risk patients.

Unlike in DLBCL or Hodgkin lymphoma, stage, erythrocyte sedimentation rate, or lactate dehydrogenase (LDH) activity, do not predict the outcome of PMBCL.^17^^,^^19^ More recently, renal involvement has been identified as a potential clinical stratification parameter, indicating a high risk of relapse, especially central nervous system (CNS) relapse in patients treated with DA-EPOCH-R.^20^

End-of-treatment 18F-fluorodeoxyglucose positron emission tomography has a high negative predictive value but only a moderate positive predictive value.^16^^,^^21^ Therefore, there is a need for stratification markers enabling to improve EFS, which is ∼10% below that observed for other types of mature B-cell non-Hodgkin lymphoma (B-NHL).^22^

Correlation of initial cytokine concentrations with patients’ characteristics could allow for the identification of patterns that are associated with disease biology, tumor burden, and relapse risk and that might be suitable as potential biomarkers for disease monitoring. We therefore investigated pretreatment plasma cytokine concentrations in a large population–based cohort of children and adolescents with PMBCL.

## Patients and methods

### Patient cohort

Sixty-two patients with reference pathology–confirmed PMBCL enrolled in the German population–based NHL-Berlin Frankfurt Münster (BFM) registry (2012 to May 2025) or the B-NHL BFM 04 trial (2005-2012, DRKS00009436), with plasma samples collected at the time of diagnosis were included. In addition, we included 9 patients with plasma samples collected at the time of relapse. Informed consent was obtained from all participants or their legal guardians. Blood plasma samples from 13 young, healthy adults were used as controls. To account for a bias introduced by long storage time, age, and sex, age-matched control plasma samples were obtained from 13 pediatric patients in long-term remission of ALK-positive ALCL, registered to the NHL-BFM registry.

### Clinical data

Staging was performed according to St. Jude^23^ and International Pediatric non-Hodgkin Lymphoma Staging System.^24^ In addition, the Ann Arbor classification was used because it allows a more granular stratification in patients with mediastinal tumors compared with conventional pediatric NHL staging. Clinical condition at diagnosis was encoded in the following 3 categories: “normal activity or slight impairment,” “moderate impairment,” and “severe impairment or critically ill.” Mediastinal tumor volume (MTV) was approximated by $V=\frac{X\times Y\times Z}{2}$.

### Cytokine measurement

All samples were processed and stored at −80°C at the central NHL-BFM laboratory. Cytokine concentrations were determined in plasma samples for the following analytes: CCL11 (eotaxin), CCL17 (TARC), CCL2 (monocyte chemoattractant protein 1), CCL3 (macrophage inflammatory protein 1α [MIP-1α]), CCL4 (MIP-1β), CCL20 (MIP-3α), CXC chemokine ligand 1 (CXCL1; GRO-α), CXCL5 (ENA-78), CXCL8 (interleukin-8 [IL-8]), CXCL9 (MIG), CXCL10 (IP-10), CXCL11 (I-TAC), IL-2, IL-4, IL-5, IL-6, IL-9, IL-10, IL-13, IL-17A, IL-17F, IL-22, interferon gamma (IFN-γ), and tumor necrosis factor α (TNF-α). The analyses were performed using the LEGENDplex Human Th Cytokine Panel and the Human Proinflammatory Chemokine Panel 1 (BioLegend, San Diego, CA), following the manufacturer’s protocols. Measurements and standard curves were performed in duplicate, and the mean values were used for downstream analysis. Concentrations were quantified on a MACSQuant 10 Flow Cytometer (Miltenyi Biotec, Bergisch Gladbach, Germany) and calculated using the LEGENDplex Data Analysis Software Suite (version 2025-05-01; Qognit, San Diego, CA).

### Statistical methods

Measurements falling below the limit of detection were imputed as 0 for downstream analysis. For graphical representation, the values were log_10_-transformed after adding a constant offset of 0.1 pg/mL to all concentrations. Statistical calculations were performed using the R statistical package (version 4.5.2, R Foundation for Statistical Computing, Vienna, Austria). Cytokine levels are reported as median values and were compared between different groups according to diagnosis, clinical, and biological characteristics using the Wilcoxon and Kruskal-Wallis tests. Correlations were calculated using the Spearman rank coefficient. The level of statistical significance was set at 0.05. Survival analysis was performed for a subgroup of patients uniformly treated with DA-EPOCH-R. EFS was defined as the time from diagnosis to relapse or progression, secondary malignancy, or death from any cause. The estimates of EFS were performed using the Kaplan-Meier method and Cox regression analysis. For this analysis, cytokine concentrations were imputed as the z-scaled natural logarithm of the measured result. Differences between groups were compared by the log-rank test.

## Results

### Patient characteristics

Clinical characteristics of the patient cohort are summarized in Table 1, and details on the control cohorts are reported in supplemental Table 1. The median age of the PMBCL cohort at diagnosis was 16.6 years (range, 5.4-21.6), and 55% of the patients were female. The age-matched group of patients with long-term remission of other types of lymphoma had a median age of 16.4 years (range, 14.1-19.9), and 53.8% (7 patients) were female. The median age of the healthy cohort was 23 years (range, 22-24), and 31% were female. Ann Arbor stages were I or II in 32 (55%), III/IV in 26 (45%), and unknown in 4 of the patents with PMBCL. Fifty-three of 57 (93%) patients had elevated LDH levels at diagnosis.

**Table 1.** **Clinical characteristics of the patient cohort**

	Initial	Relapse/progression
Cohort, n∗	62	9
Age, mean (min-max), y	16.2 (10.3-21.7)	15.1 (8.4-17.1)
**Sex**		
Male	28 (45)	4 (44)
Female	34 (55)	5 (56)
**Stage (Murphy)**		
III	60 (98)	8 (89)
IV	0	1 (11)
Not possible†	1 (2)	0
Not available	1	0
**Stage (Ann Arbor)**		
II	32 (55)	5 (56)
III	3 (5)	3 (33)
IV	23 (40)	1 (11)
Not available‡	4 (7)	—
LDH, mean (min-max), U/L	553 (204-1322)	Not available
**Frontline treatment**		
DA-EPOCH-R	50 (81)	9 (100)
B-NHL04	9 (15)	0
Other	3 (4.8)	0
**Outcome**		
FOP	50 (81)	7 (78)
Progression/relapse	12 (19)	0
Death	0	2 (12)
**B symptoms**		
Yes	28 (45)	—
No	27 (44)	—
Not assessed or available	7 (11)	9 (100)
**Manifestations**		
Mediastinal	62/62 (100)	8 (89)
Pleural effusion	27/56 (48)	0
Pericardial effusion	27/58 (47)	0
Lung	17/56 (30)	0
Subdiaphragmatic	17/58 (29)	3 (33)
Renal	8/62 (13)	1 (11)
Liver	3 (5)	0
CNS	0	1 (11)
Bone marrow	0	0

Data are presented as number (%) unless otherwise specified.

FOP, freedom of progression; max, maximum; min, minimum.

∗ From 4 patients, the samples were available both at initial diagnosis and in relapse.

† In 1 patient, no initial bone marrow aspiration and lumbar puncture was performed; therefore, the stage is not available.

‡ Details on manifestations were not reported; therefore, Ann Arbor stage could not be assessed.

Eleven patients, diagnosed before 2012, received treatment according to the B-NHL04 trial.^17^ Fifty-one of 62 patients were treated with DA-EPOCH-R. In the latter group, the median follow-up time was 2.4 years, and the EFS at 2 years was 86% ± 5%. No patient of the initial cohort died.

### Preanalytical factors associated with plasma cytokine concentrations

Of the 62 samples at initial diagnosis, 34 were collected from the peripheral blood and 28 from the bone marrow. The concentrations of CXCL10 were lower in the bone marrow samples (Wilcoxon; *P* = .02). To assess the potential impact of the storage time, a correlation analysis was performed, which indicated that CXCL1 and CXCL5 concentrations increased during long-term storage, with a correlation coefficient >0.4 (supplemental Figure 1). These chemokines were therefore excluded from further analyses.

### Initial plasma cytokine concentration of patients with PMBCL

The concentrations of CCL4, CCL17, CXCL8, CXCL9, CXCL10, CXCL11, and IL-6 were significantly higher in the plasma of patients at PMBCL diagnosis than in both healthy controls and age-matched patients in remission of ALK-positive ALCL (Figure 1; supplemental Table 2). The concentrations of IL-10 were only significantly higher in patients with PMBCL compared with healthy controls; the concentrations of CCL2, CCL11, and CCL20 were only significantly higher in patients with PMBCL compared with patients in remission of ALK-positive ALCL. The concentrations of IL-5 were lower in patients at PMBCL diagnosis compared to those in remission of ALK-positive ALCL.

**Figure 1.**
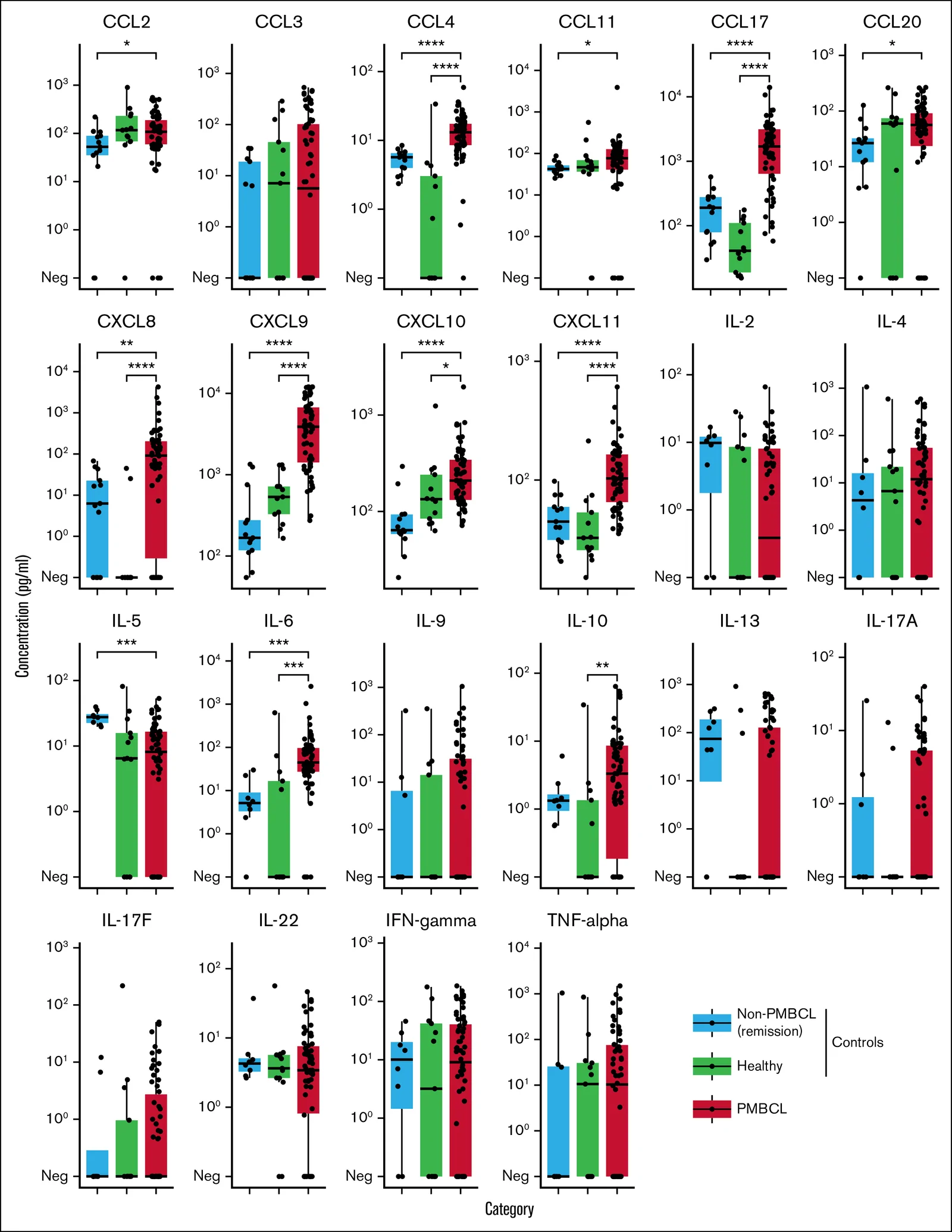
**Blood cytokine concentrations at initial diagnosis of patients with PMBCL compared with controls.** Cytokine concentrations in patients with PMBCL at diagnosis (n = 62), age-matched patients with ALK-positive ALCL in long-term remission (n = 13), and healthy young adults (n = 13). With the cytokine panel (interleukins, IFN-γ, and TNF-⍺), only 8 samples of age-matched controls could be analyzed. Logarithmic representation, with values in picogram per mL. Statistical significance is denoted by asterisks (∗*P* < .05; ∗∗*P* < .01; ∗∗∗*P* < .001; ∗∗∗∗*P* < .0001). Neg, negative.

### Association of cytokine concentrations with initial clinical characteristics

The concentrations of IL-4 (25.4 pg/mL vs 5.13 pg/mL; *P* = .024) and IL-9 (15.5 pg/mL vs 0 pg/mL; *P* = .047) were higher in male than in female patients (supplemental Figure 2). No significant correlations were observed between the plasma cytokine concentrations and the age of patients at diagnosis (supplemental Figure 3). The concentrations of IL-17F and TNF-α were lower in patients who presented with B symptoms compared with those without B symtoms (median, 0 vs 0.54 pg/mL; *P* = .015 and median, 0 vs 36.6 pg/mL; *P* = .035, respectively; supplemental Figure 4). IL-5 (*P* = .001), IL-13 (*P* = .009), IL-22 (*P* = .032), IFN-γ (*P* = .008), CCL3 (*P* = .021), and CCL20 (*P* = .004) levels were positively correlated with better general condition at the time of diagnosis (supplemental Figure 5).

There was no correlation of initial cytokine concentrations with Ann Arbor stage (supplemental Figure 6A). The concentrations of CXCL9, CXCL10, CXCL11, and CCL17 correlated positively with LDH activity at diagnosis in 57 patients with available data (Figure 2B). The concentrations of IL-17F and IL-22 were inversely correlated with LDH activity. An approximated MTV was available in 35 patients and correlated positively with the concentration of CCL17 (*R* = 0.43; *P* = .037). A significant inverse correlation was observed for IL-13 (*R* = −0.5; *P* = .002), IL-5 (*R* = −0.6; *P* < .001), IL-17F (*R* = −0.46; *P* = .006), and IL-22 (*R =* −0.52; *P* = .001) with MTV. There was a trend for an inverse correlation for IL-17A level (*R* = −0.31, *P* = .065). There was no association of cytokine concentrations with subdiaphragmatic manifestations, which were observed in 17 of 58 (29%) patients. Eight patients with renal involvement had lower concentrations of CCL20 compared with patients without renal involvement (27.7 pg/mL vs 63.4 pg/mL; *P* = .028; supplemental Figure 6B). Patients with pericardial and/or pleural effusion had significantly lower median concentrations of IL-4, IL-5, IL-17A, IL-22, IFN-γ, and TNF-α compared with those without pericardial and/or pleural effusion (supplemental Figure 6).

**Figure 2.**
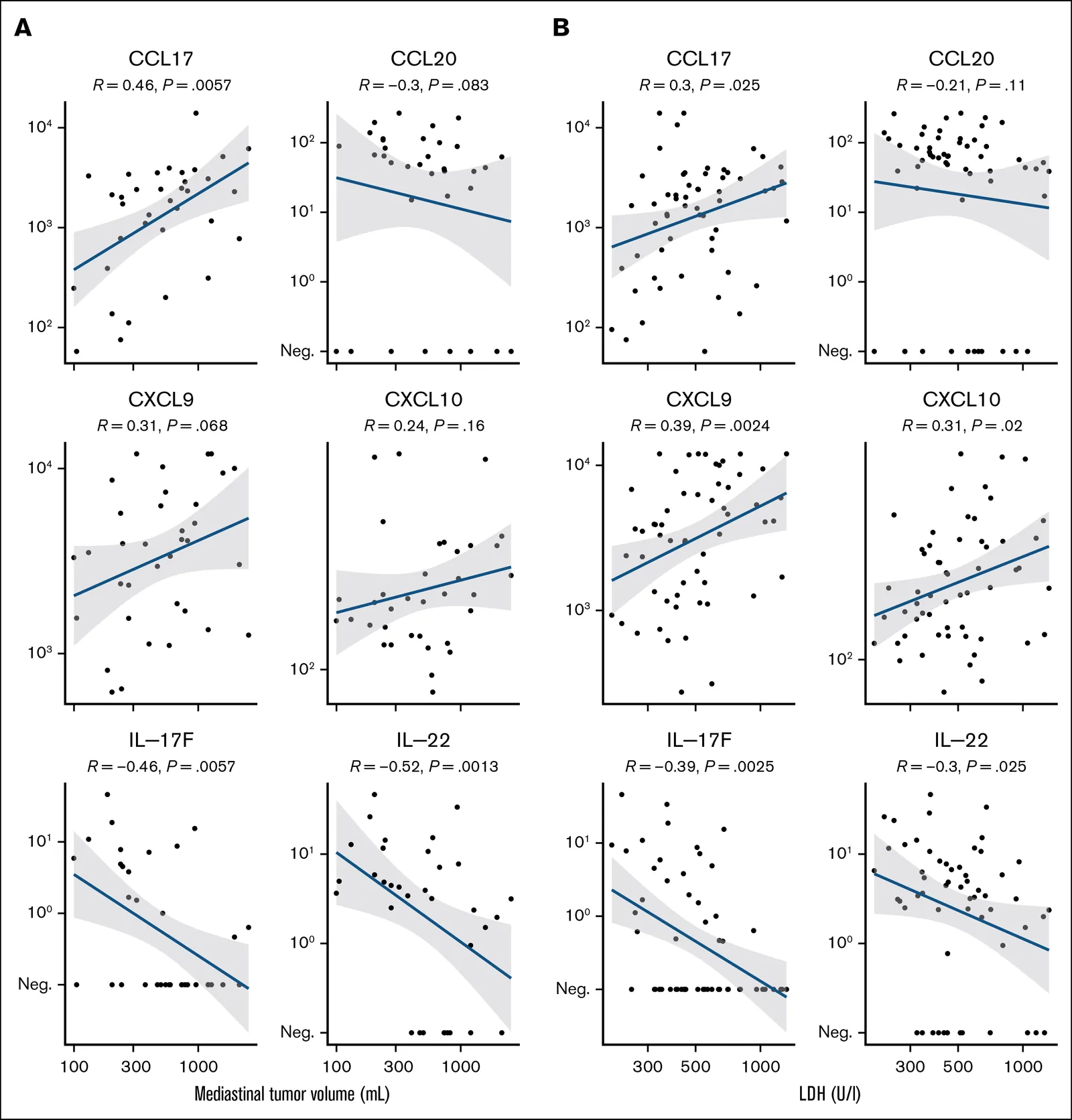
**Blood cytokine concentrations in relation to mediastinal tumor volume and LDH.** Correlation of cytokine levels with MTV (mL) (A) in 35 patients and with LDH (U/L) (B) in 57 patients. Only selected cytokines of interest are shown; cytokine concentrations are presented on a logarithmic scale in pg/mL. The Spearman rank correlation coefficients and *P* values are shown.

### Patterns and correlation between cytokines

A correlation analysis (supplemental Figure 7) to identify cytokine patterns at initial diagnosis showed high correlations among IL-4, IL-17A, IL-17F, IL-22, and TNF-α levels (with correlation coefficients between 0.7 and 0.9; *P* < .001) and between CCL11 and CXCL11 concentrations (*R* = 0.7; *P* < .001). A moderate correlation was observed between IL-6 and IL-10 and between IFN-γ–induced CXCL9, CXCL10, and CXCL11. An unsupervised clustering and heat map of normalized cytokine concentrations (Figure 3) broadly recapitulated function annotation: elevated concentrations of most CCL chemokines were characteristic of the whole cohort. T helper 1 cell (Th1)–, Th2-, and Th17-associated markers were elevated in a subset of patients with high levels of IL-2, IL-4, IL-9, IL-17A, IL-17F, IL-22, and TNF-α.

**Figure 3.**
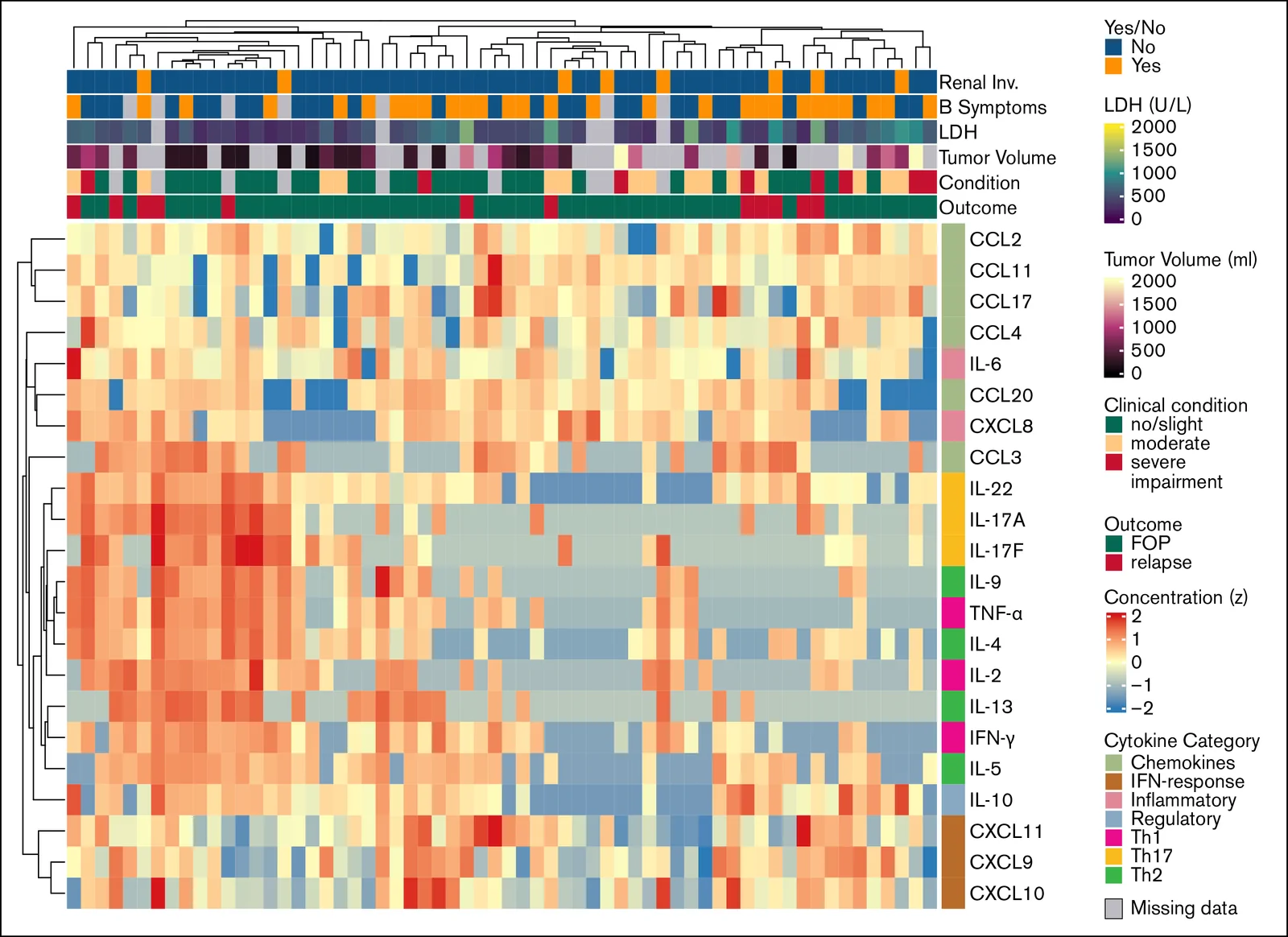
**Heat map of normalized cytokine concentrations.** An unsupervised clustering and heat map of the z-scaled log-transformed cytokine concentrations annotated with clinical characteristics (renal involvement, B symptoms, general conditions, LDH level, MTV, and outcome). Cytokines are broadly categorized by functional roles (supplemental Table 3). FOP, freedom of progression.

### Associations of plasma cytokine concentrations with outcome

For outcome analysis, we excluded 12 patients who received BFM-type therapy without rituximab, leaving 50 patients uniformly treated with DA-EPOCH-R, of whom 6 (12%) experienced a relapse. Univariate Cox regression analysis showed a negative association of IL-6, IL-10, IL-17A, and IL-22 with EFS, with hazard ratios of 6.8 (95% confidence interval [CI], 1.5-30.9), 6.6 (95% CI, 1.5-28.4), 2.7 (95% CI, 1-6.9), and 4.5 (95% CI, 1.1-19.1), respectively (Figure 4A). Multivariate analysis was not performed due to the low number of events. The concentrations of IL-10 (27.5 pg/mL vs 6.7 pg/mL; *P* = .006), IL-17A (5.2 pg/mL vs 0 pg/mL; *P* = .025), and IL-22 (11.6 pg/mL vs 3.1 pg/mL; *P* = .013) were significantly higher in initial samples of patients who later experienced a relapse (Figure 4B). Patients with concentrations at or above the upper quantile of IL-6 (*P* = .012), IL-10 (*P* = .012), and IL-22 (*P* = .03) had inferior EFS by comparison with log-rank test (Figure 4C-E). For IL-17A level, no significant difference in EFS was observed.

**Figure 4.**
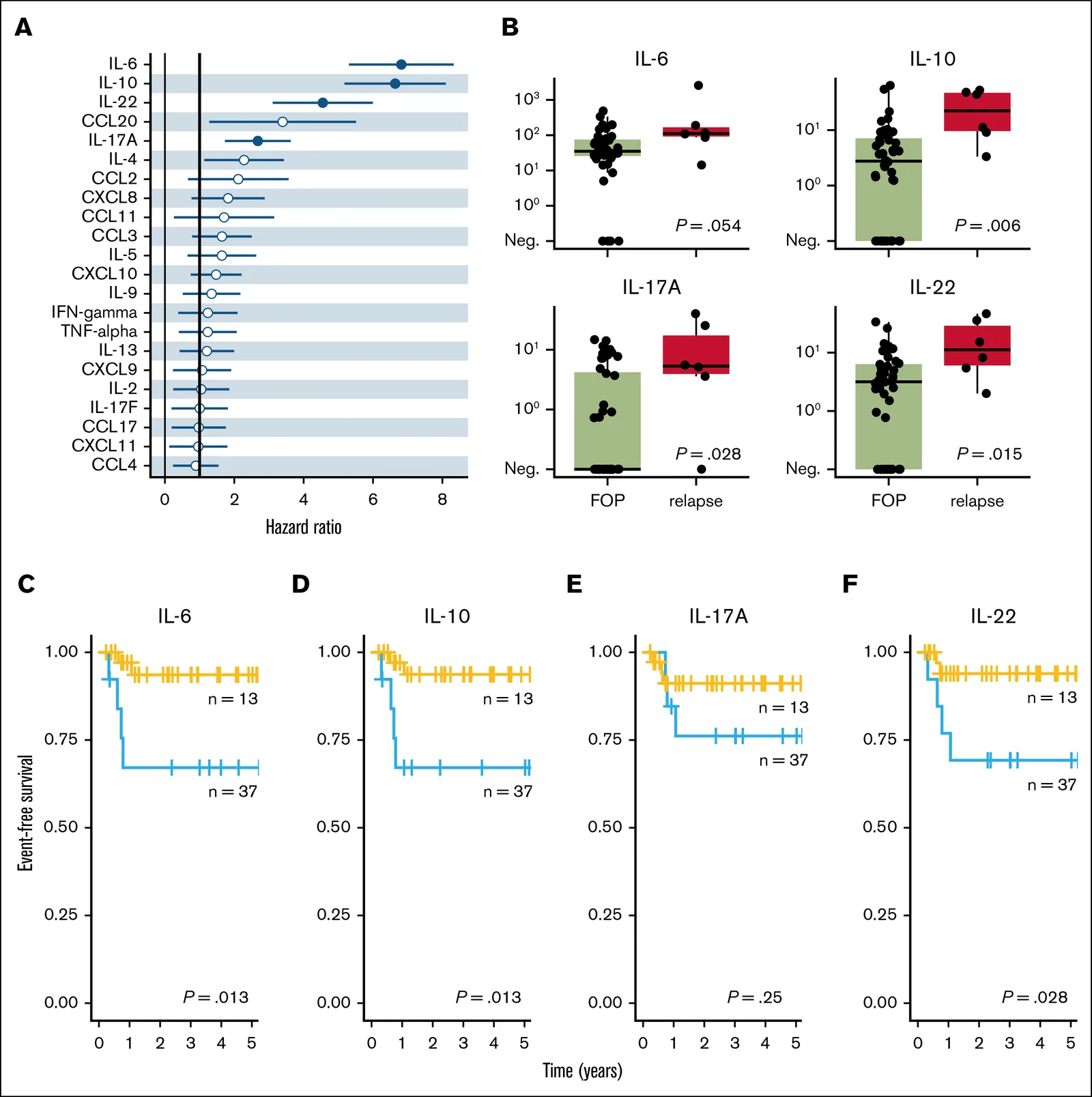
**Outcome and initial cytokine concentrations.** Univariate Cox regression analysis for EFS with the z-scaled natural logarithm of the initial cytokine concentrations as parameter. For this analysis, only patients uniformly treated with DA-EPOCH-R were included. (A) Hazard ratios with 95% CIs are shown, with the black dots representing significant findings. (B) Elevated initial cytokine concentration in patients with later relapse. (C-F) EFS by initial cytokine concentration above (n = 13) or below (n = 37) the upper quartile in cytokines with significant results in the univariate Cox regression analysis.

### Cytokine concentrations during treatment and at relapse

In 3 patients with measurements after initial diagnosis, the concentrations of initially elevated cytokines generally declined during frontline treatment (supplemental Figure 8). In 9 patients, samples were available at time of relapse. Significantly, IL-2 and CCL3 were not detectable in patients at relapse, compared to 32 of 62 and 30 of 62 at initial diagnosis, respectively. The median concentrations of CXCL9 were significantly lower at relapse than at initial diagnosis (965.6 vs 3883.5 pg/mL; *P* = .017; supplemental Figure 9). CCL17 concentrations at relapse were comparable to initial diagnosis. In 1 patient, CCL17 level was not elevated at suspicion of relapse but increased during diagnostic workup and the start of second-line treatment (from 40.7 pg/mL to 111.8 pg/mL and 304 pg/mL after 6 and 31 days, respectively). Another patient with isolated CNS relapse had a CCL17 concentration comparable to healthy controls (supplemental Figure 10).

## Discussion

Approximately 85% of patients with PMBCL are cured by dose-intensive chemoimmunotherapy.^25^ Currently, the lack of validated clinical risk factors and difficulty of monitoring the treatment response by imaging are the main obstacles in improving patient outcomes. Integrating cytokine profiling may provide deeper insight into disease biology and could help to identify a disease-specific cytokine signature in PMBCL and might serve as prognostic parameter.

In PMBCL, genetic studies and in vitro models demonstrated CCL17 as a hallmark cytokine.^11^^,^^12^ CCL17 is elevated in Hodgkin lymphoma,^13^^,^^26^^,^^27^ a disease sharing features with PMBCL. Genetic aberrations in PMBCL, such as *REL* amplifications, result in the strong upregulation of CCL17 in cell lines.^11^ CCL17 recruits regulatory T cells via CCR4^28^ and may be involved in the thymic tropism of PMBCL.^12^ CCL17 level was elevated in most patients at diagnosis, correlated with LDH activity and MTV, but was not associated with the risk of relapse. Its suitability as a biomarker for disease monitoring might be limited based on the observation that CCL17 level was not elevated in 1 patient with CNS relapse and at initial clinical suspicion of relapse in another patient.

Both proinflammatory and immunoregulatory cytokine levels are elevated in the blood of patients with lymphoma, including ALCL,^6^^,^^29^ Hodgkin lymphoma,^30^^,^^31^ and DLBCL.^32^^,^^33^ Most interleukins and several chemokines exert pleiotropic effects through tyrosine kinases such as JAKs and their downstream transcription factors, STAT.^34^ The JAK/STAT pathway is altered in PMBCL, primarily by the overactivation of JAK2/STAT6 signaling.^1^^,^^10^^,^^35^

In our cohort, elevated IL-6, IL-10, IL-17A, and IL-22 levels were associated with inferior EFS following uniform treatment with DA-EPOCH-R. IL-6 and IL-10 levels showed no association with tumor burden or baseline clinical features, whereas IL-17A and IL-22 levels were correlated inversely with tumor burden. This finding is consistent with previous reports indicating the prognostic relevance of factors other than tumor burden.^9^^,^^36^ The proinflammatory cytokine IL-6 is associated with adverse outcomes across various types of cancers.^37^ IL-10 contributes to the pathogenesis of ALCL^6^^,^^38^ and in Hodgkin lymphoma, Reed-Sternberg cells produce IL-10, contributing to the immune-suppressive microenvironment.^31^ The constitutive activation of NF-κB pathways in DLBCL induces the expression of IL-6 and IL-10.^39^

IL-6, IL-10, and IL-22, which shares similarities with IL-10,^40^ mediate signaling through JAK1/JAK2 and STAT3,^41^^,^^42^ potentially supporting disease dissemination by increasing cell mobility in DLBCL^43^ or upregulation of PD-L1 expression.^44^^,^^45^ IL-17A signals via Act1/TRAF6 instead of the JAK/STAT pathways.^46^ Its pleiotropic downstream effects include the upregulation of cytokine expression, including IL-6^47^; however, no correlation between these cytokines was observed in our cohort.

The plasma cytokine profiles at diagnosis did not match to a clearly polarized T helper subset. However, cytokines associated with Th1, Th2, and Th17 responses were positively correlated at baseline, suggesting a T-cell–mediated immune response. Further investigation of the PMBCL microenvironment is warranted to identify the cellular sources of elevated cytokine levels.

The fact that the concentrations of IFN-γ–regulated cytokines CXCL9 and CXCL10 were markedly elevated at diagnosis and correlated with LDH activity and tumor volume, could also hint toward a Th1-polarized immune response and upregulation of IFN-γ because these downstream cytokines are more stable than IFN-γ itself.^48^^,^^49^

Renal involvement has emerged as a potential clinical risk factor.^20^^,^^50^^,^^51^ In our study, renal involvement was associated with lower concentrations of CCL20 compared with those in patients without renal involvement. CCL20 is a chemotactic ligand of CCR6, expressed on Th17 and regulatory T cells^52^ with CCL20/CCR6 promoting immune escape, as shown for Hodgkin lymphoma.^53^ From our observations, the role of reduced CCL20 in patients with renal involvement remains unclear.

Our study has several limitations, including preanalytical influences, which we addressed by excluding CXCL1 and CXCL5 from further analyses because these cytokines showed significant correlations with storage time, potentially explained by release from degrading residual platelet granules.^54^ Although our cohort represents a large population–based pediatric cohort, the number of patients especially in subgroups is low, an inherent limitation in rare diseases such as PMBCL. Our cohort included both peripheral blood– and bone marrow–derived plasma samples; however, considering that only CXCL10 concentration showed a significant difference between these 2 sample types, we decided to include both in the analysis. Given the limited number of events in our cohort, the statistical analysis for EFS should be interpreted with caution and requires validation in larger, independent cohorts. Cytokine expression patterns in the peripheral blood are only partly representative of the tumor microenvironment where they often are produced by tumor, immune, or stromal cells. The complications of the tumor, such as infections or thrombosis of large vessels, could also modulate cytokine production.

Although we only analyzed pediatric and adolescent patients with PMBCL, the lack of an observed association between cytokine levels and age may be consistent with the notion of PMBCL as a disease spanning the adolescent and young adult age spectrum.

In summary, our findings suggest that the expression of IL-6, IL-8, IL-10, CCL4, CCL17, CXCL9, and CXCL10 forms a typical cytokine signature for PMBCL, with a potential prognostic value for IL-6, IL-10, IL-17A, and IL-22. Further prospective evaluation is required to establish CCL17 as a potential parameter for treatment monitoring and to correlate cytokine measurements with underlying differences in the molecular biology of PMBCL.

Conflict-of-interest disclosure: The authors declare no competing financial interests.

## Supplementary Material

Supplemental Tables, Figures, and References
