# Supplementary material for: Cytokine concentrations in pediatric primary mediastinal large B-cell lymphoma correlate with disease extent and outcome
Source: Blood Adv. 2026 Sep 12;10(22):7525–35. doi: 10.1182/bloodadvances.2026021463 (PMC13770619; doi:10.1182/bloodadvances.2026021463)
Supplement: Supplementary file 1 [file BLOODA_ADV-2026-021463-mmc1.pdf]

Supplementary Appendix

Cytokine concentrations in pediatric primary mediastinal large B cell lymphoma correlate with disease extent and outcome

Table of contents

Supplementary Tables 2
Supplementary Table 1: Clinical characteristics of comparison cohort 2
Supplementary Table 2: Cytokine concentrations of PMBCL patients and in controls 3
Supplementary Table 3: Functional Categorization of analyzed cytokines and chemokines 4

Supplementary Figures 5
Supplementary Figure S1: Correlation of cytokine levels with storage time. 5
Supplementary Figure S2: Differences in cytokine concentrations by sex 6
Supplementary Figure S3: Correlation of cytokine concentrations and age 7
Supplementary Figure S4: Cytokine concentrations by the presence of B symptoms 8
Supplementary Figure S5: Cytokine concentrations by general condition 9
Supplementary Figure S6: Cytokine concentration by stage, effusions and renal involvement ... 10
Supplement Figure S8: Longitudinal changes in cytokine concentrations during therapy 12
Supplement Figure S9: Cytokine concentrations at initial diagnosis and at relapse 13
Supplementary Figure S10: CCL17 levels at initial diagnosis, during treatment and at relapse .. 14

References 15

Supplementary Tables

Supplementary Table 1: Clinical characteristics of comparison cohort

Clinical characteristics are presented for an age-matched comparison group of ALCL patients in remission and a cohort of healthy young individuals.

	**Age Matched**	**Controls**
	Remission	Healthy
**N**	13	13
**Diagnosis**	ALK-positive ALCL	-
**Age**<br>mean (min, max)	16.9 (14.1-19.9)	22.8 (21-26)
**Sex**		
Male	5 (38%)	9 (69%)
Female	8 (62%)	4 (31%)
**Years since diagnosis**<br>mean (min, max)	5.53 (3.26 – 12.07)	-
**Years since end of treatment**<br>mean (min, max)	5.16 (2.8-11.7)	

Supplementary Table 2: Cytokine concentrations of PMBCL patients at diagnosis and in controls

With the cytokine panel (Interleukins, IFN-γ, and TNF-α), only 8 samples of the age matched control group could be analyzed. SD, standard deviation.

Analyte	Age Matched in Remission		Healthy Young Adults		PMBCL at Diagnosis		p-value (Kruskal)
	Detectable n (%)	Mean±SD (pg/mL)	Detectable n (%)	Mean±SD (pg/mL)	Detectable n (%)	Mean±SD (pg/mL)	
CCL2	11 (85%)	67±58	12 (92%)	188±232	59 (95%)	145±134	**0.028**
CCL3	6 (46%)	9±13	7 (54%)	54±91	32 (52%)	84±139	0.4
CCL4	13 (100%)	5±2	6 (46%)	4±9	61 (98%)	14±9	**<0.001**
CCL11	13 (100%)	47±17	11 (85%)	95±149	57 (92%)	146±487	**0.036**
CCL17	13 (100%)	201±155	13 (100%)	66±55	62 (100%)	2455±2834	**<0.001**
CCL20	12 (92%)	32±35	9 (69%)	67±81	50 (81%)	71±66	0.13
CXCL8	9 (69%)	18±22	2 (15%)	5±14	46 (74%)	256±643	**<0.001**
CXCL9	13 (100%)	375±445	13 (100%)	634±409	62 (100%)	4681±3668	**<0.001**
CXCL10	13 (100%)	91±74	13 (100%)	231±313	62 (100%)	300±285	**<0.001**
CXCL11	13 (100%)	50±23	13 (100%)	50±52	62 (100%)	127±94	**<0.001**
IL-2	6/8 (75%)	8±6	6 (46%)	7±10	31 (50%)	5±10	0.3
IL-4	5/8 (63%)	140±374	8 (62%)	58±161	42 (68%)	61±124	0.6
IL-5	8/8 (100%)	28±7	9 (69%)	15±22	41 (66%)	11±12	**0.002**
IL-6	7/8 (88%)	9±11	5 (38%)	58±174	58 (94%)	132±348	**<0.001**
IL-9	3/8 (38%)	42±113	4 (31%)	32±97	29 (47%)	51±147	0.5
IL-10	8/8 (100%)	2±2	5 (38%)	3±9	46 (74%)	8±14	**0.011**
IL-13	6/8 (75%)	121±122	3 (23%)	100±258	24 (39%)	104±180	0.2
IL-17A	3/8 (38%)	4±9	2 (15%)	1±4	27 (44%)	4±7	0.2
IL-17F	2/8 (25%)	2±5	4 (31%)	17±60	27 (44%)	4±10	0.6
IL-22	8/8 (100%)	8±12	11 (85%)	8±15	47 (76%)	7±10	0.7
IFN-γ	6/8 (75%)	15±16	7 (54%)	33±54	42 (68%)	30±43	>0.9
TNF-α	3/8 (38%)	137±366	7 (54%)	84±232	34 (55%)	113±259	0.7

Supplementary Table 3: Functional Categorization of analyzed cytokines and chemokines

Cytokines and chemokines were grouped into six functional categories based on their predominant role reported in the in the literature, indicated by superscript references.

Chemokines[1]	IFN-response[2]	Inflammatory[3]	Regulatory[4]	Th1[4]	Th17[4]	Th2[4]
CCL2	CXCL9	IL-6	IL-10	IL-2	IL-17A	IL-4
CCL3	CXCL10	CXCL8		IFN-γ	IL-17F	IL-5
CCL4	CXCL11			TNF-α	IL-22	IL-9
CCL11						IL-13
CCL17						
CCL20						

Supplementary Figures

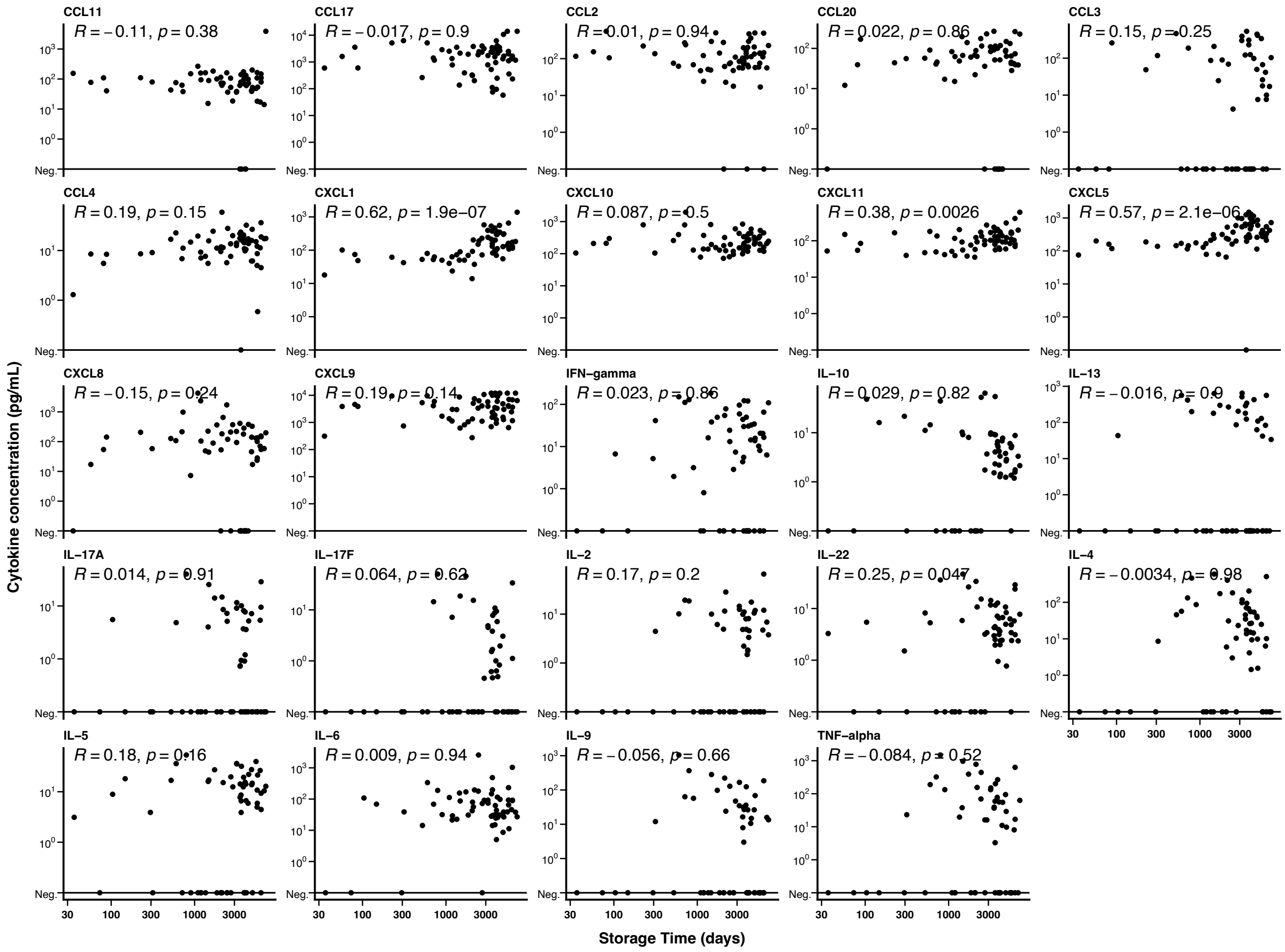

Supplementary Figure S1: Correlation of cytokine levels with storage time.

Correlation analyses between storage time in days and cytokine concentrations at initial diagnosis were performed for 24 analytes. Each dot represents an individual sample (n=62). Cytokine levels are displayed on a logarithmic scale in pg/mL. Spearman´s rank correlation coefficient and corresponding p-values are indicated in each plot.

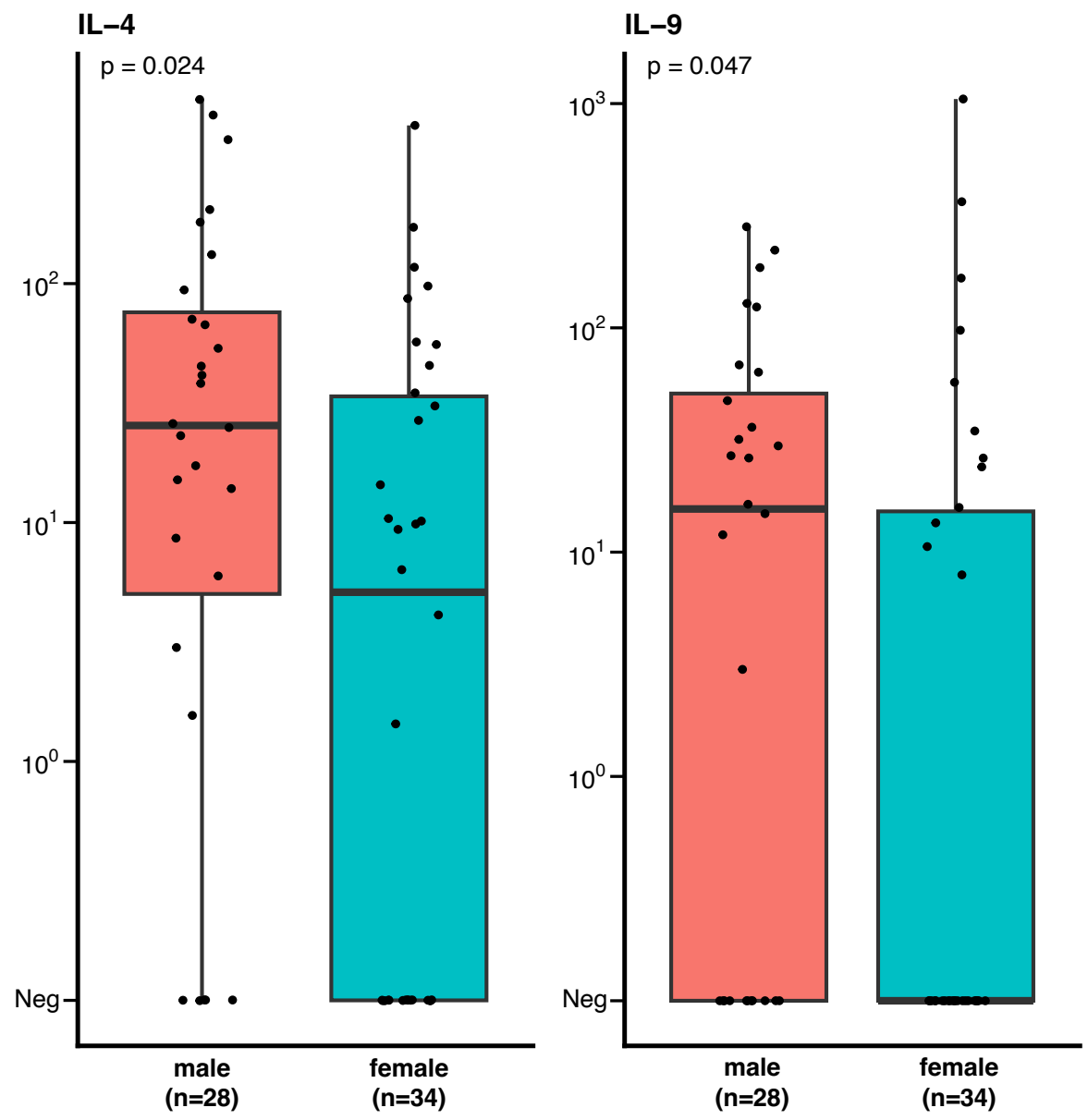

Supplementary Figure S2: Differences in cytokine concentrations by sex

Initial cytokine concentrations (pg/mL) were compared between male (n=28) and female (n=34) patients. Only IL-4 and IL-9 showed statistically significant differences. Boxplots indicate median, interquartile range, minimum and maximum. Cytokine levels are shown on a logarithmic scale. Statistical comparisons were performed using the Wilcoxon rank-sum test.

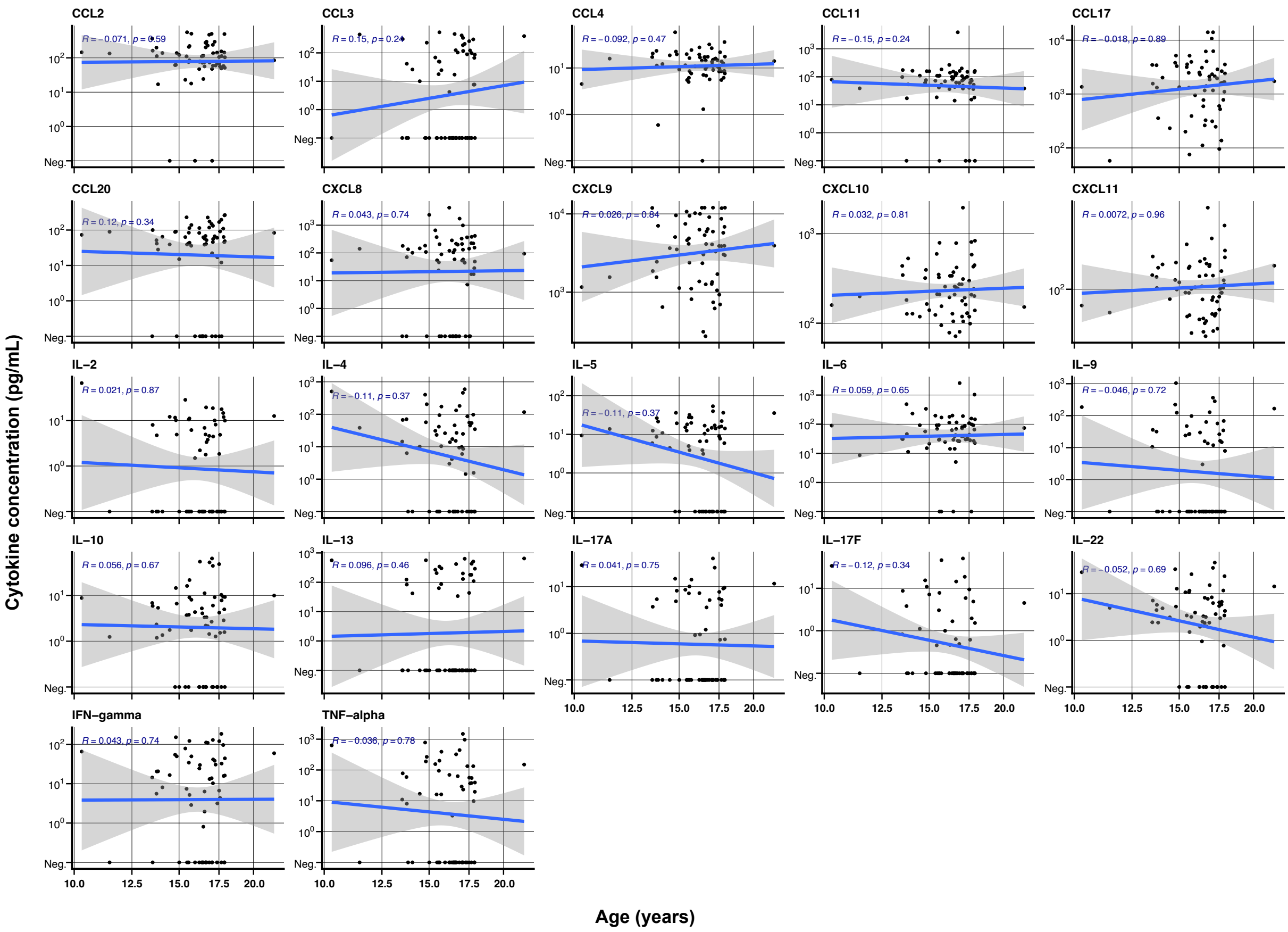

Supplementary Figure S3: Correlation of cytokine concentrations and age

Correlation analyses between patient age in years and cytokine levels (pg/mL) for 22 analytes at initial diagnosis. Spearman´s rank correlation coefficient and corresponding p-values are indicated in each plot. No significant correlation was found for any cytokine.

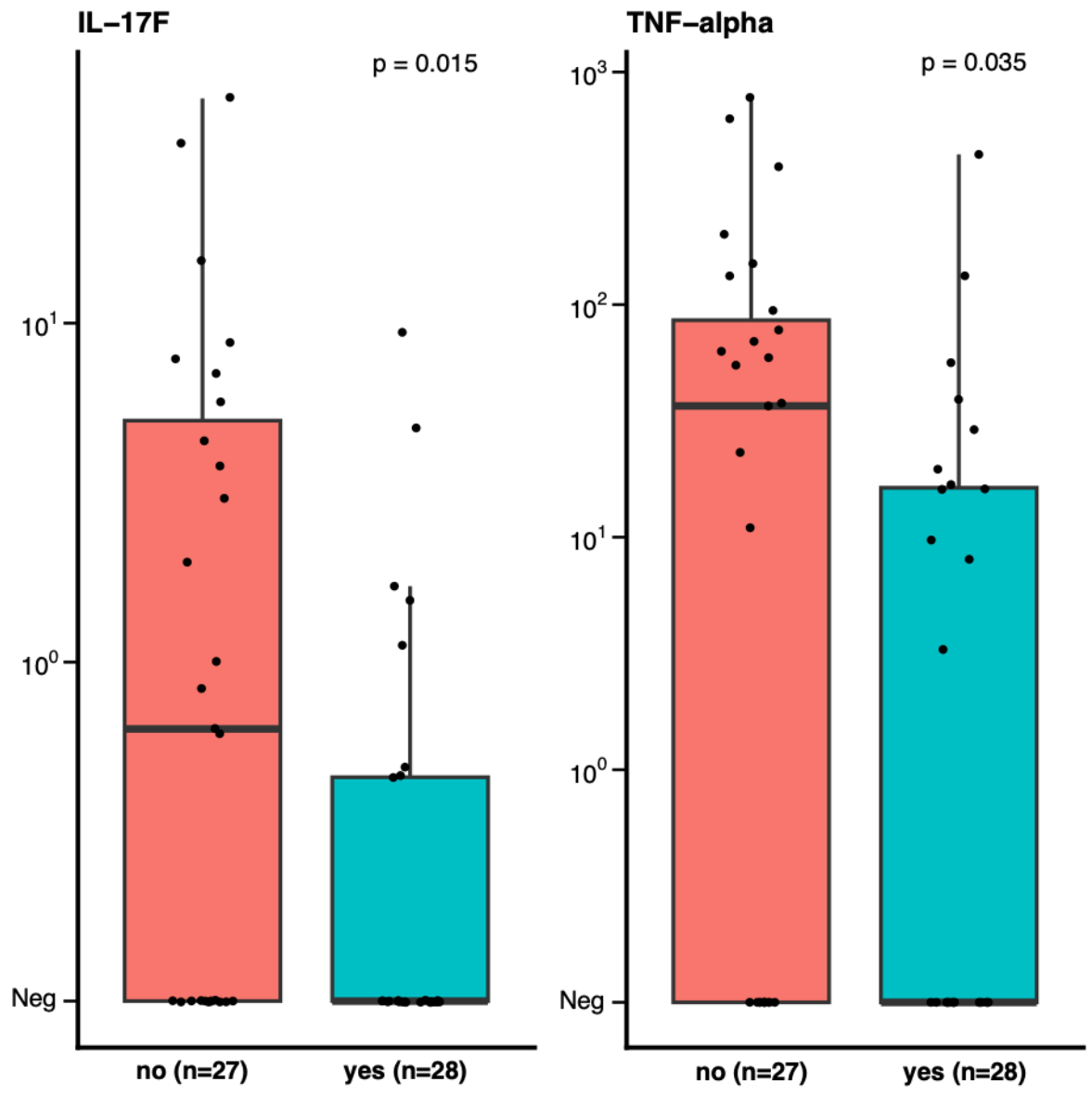

Supplementary Figure S4: Cytokine concentrations by the presence of B symptoms

Cytokine concentrations (pg/mL) were compared between patients with B symptoms (n=27) and those without B symptoms (n=28). B symptoms are defined as the presence of fever, weight loss, or night sweats. Only analytes showing statistically significant differences are displayed. Boxplots indicate median, interquartile range, minimum and maximum. Cytokine levels are shown on a logarithmic scale. Statistical comparisons were performed using Wilcoxon rank-sum test.

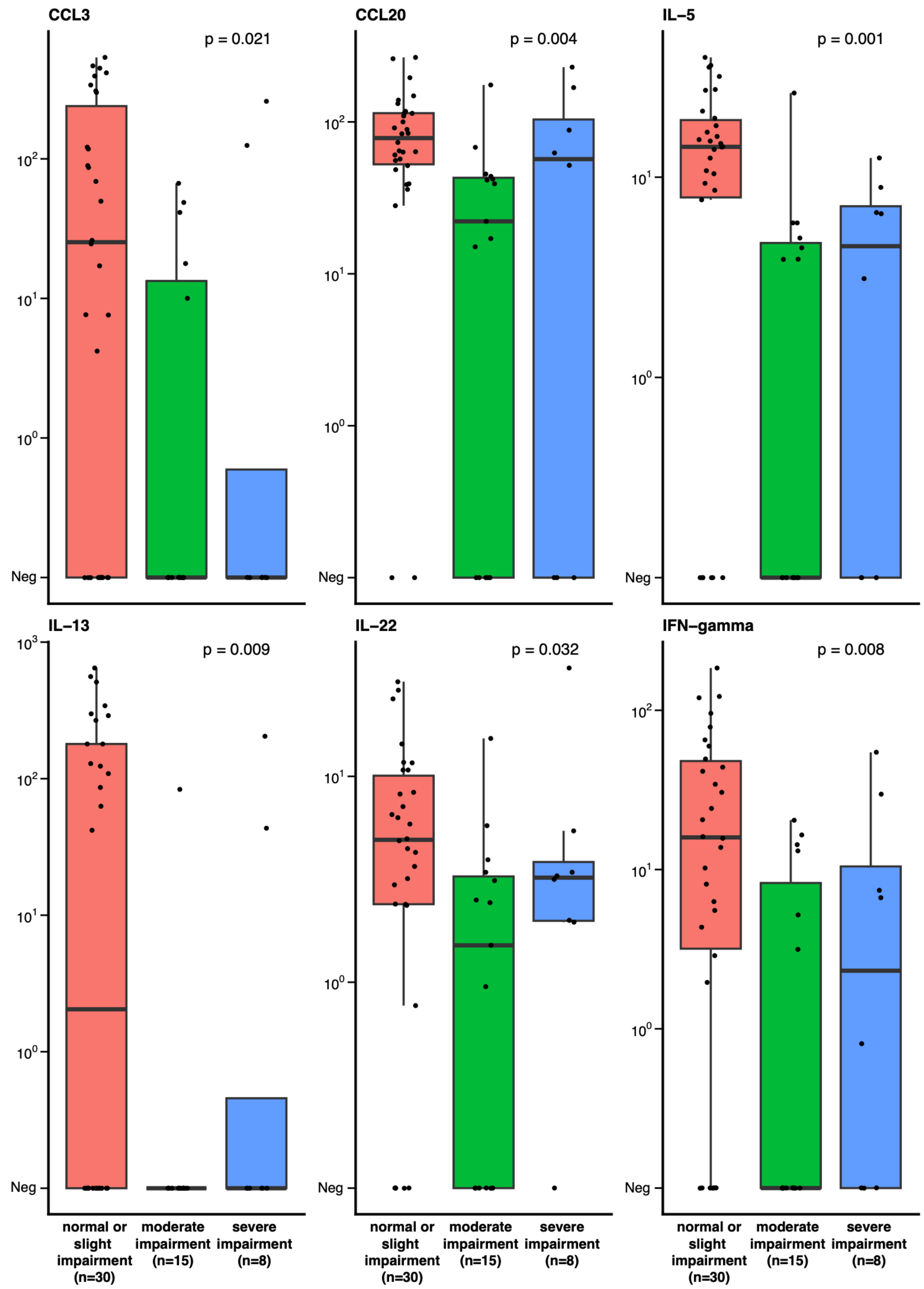

Supplementary Figure S5: Cytokine concentrations by general condition

Initial cytokine concentrations (pg/mL) were compared across three groups stratified by general condition. Each dot represents an individual sample, and boxplot show median, interquartile range, minimum and maximum. Cytokine levels are displayed on a logarithmic scale. Only analytes with statistically significant differences are shown. Statistical comparisons between groups were performed using the Kruskal-Wallis test.

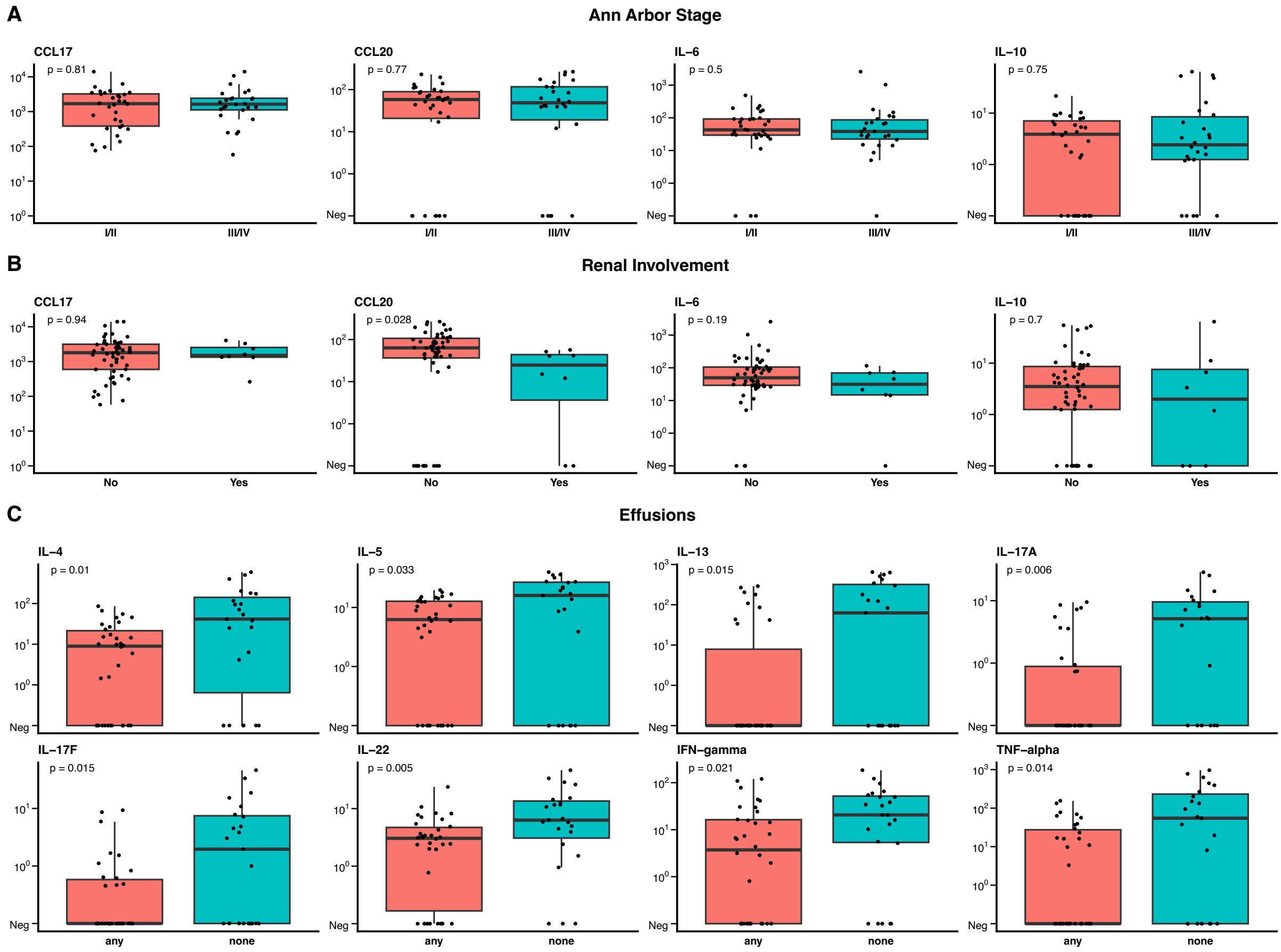

Supplementary Figure S6: Cytokine concentration by stage, effusions and renal involvement

Cytokine concentrations (logarithmic scale, pg/mL) in patients (A) with Ann Arbor stage I/II (n=32) versus stage III/IV (n=26); (B) with (n=6) renal involvement or without (n=54); (C) with (n=34) or without (n=23) pleural and/or pericardial effusions. Only selected cytokines of interest are shown for (A) and (B), for (C) all cytokines with statistically significant differences are shown. Statistical comparisons were performed using Wilcoxon rank-sum test.

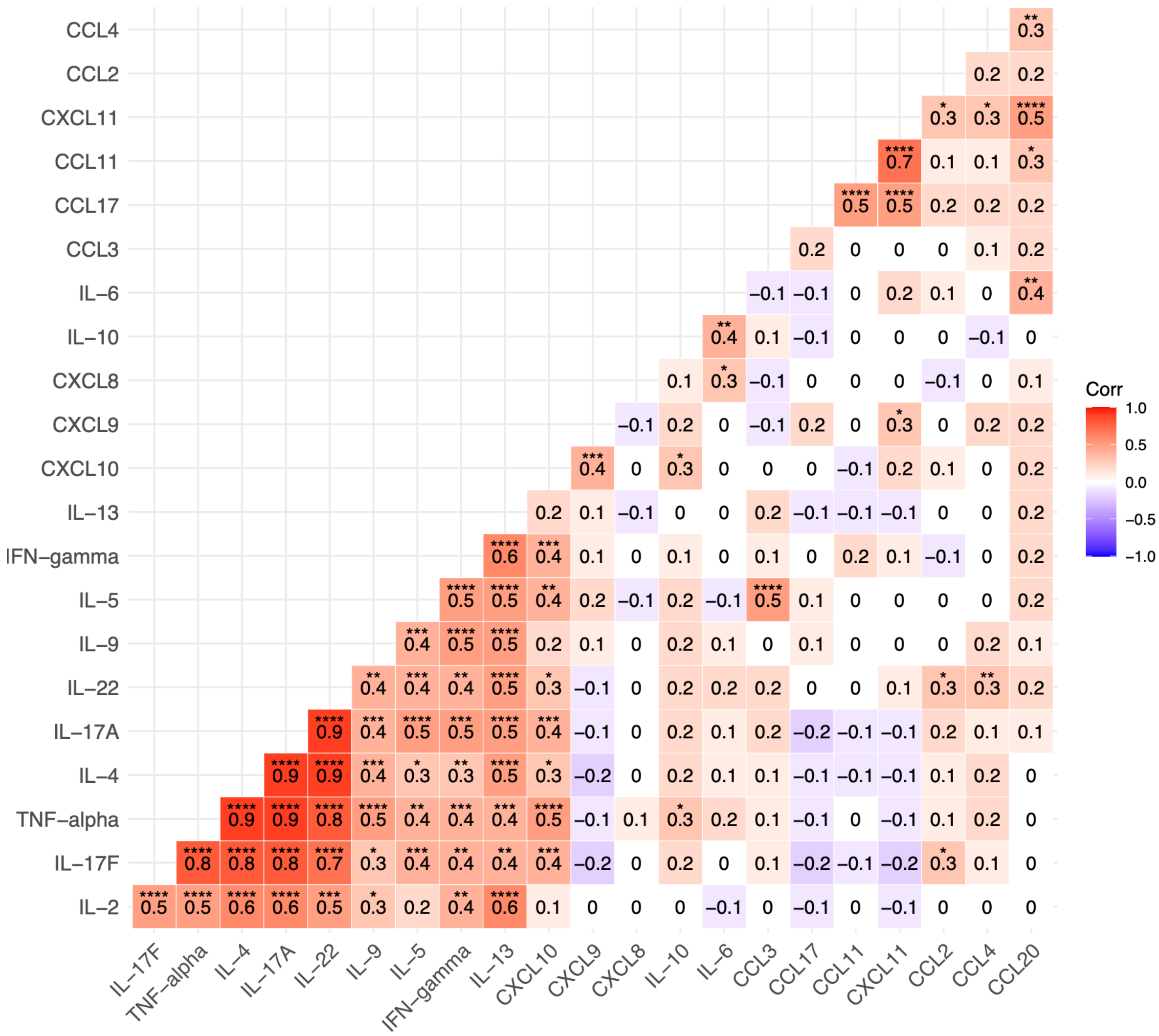

Supplementary Figure S7: Pairwise correlation of cytokines at initial diagnosis

Each square represents the Spearman´s rank correlation coefficient for the respective cytokine pair, with correlation coefficients numerically within each square. The strength and direction of correlations are indicated by the color scale. Statistical significance is denoted by asterisks (* p<0.05, ** p<0.01, *** p<0.001, **** p<0.0001).

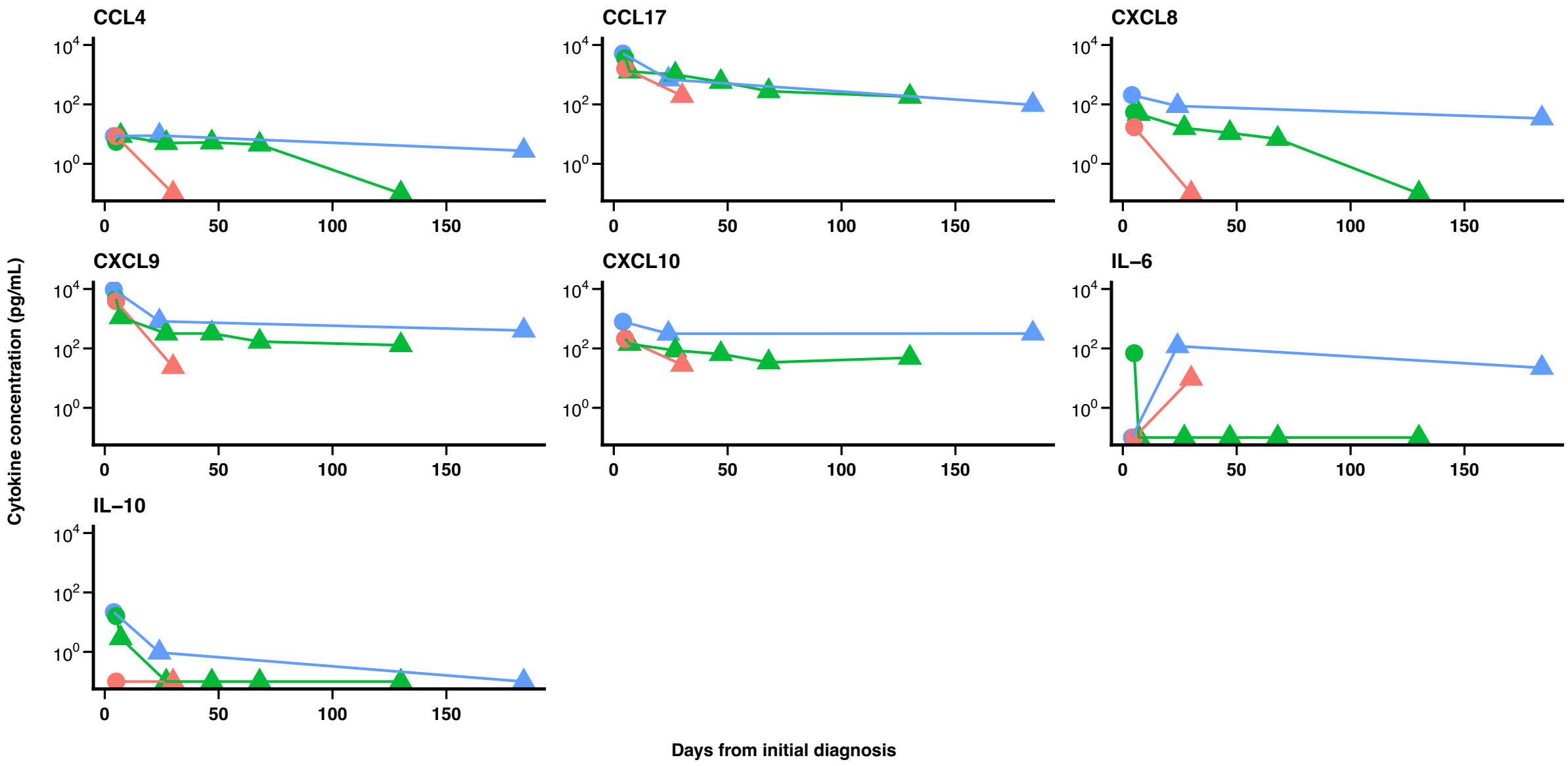

Supplement Figure S8: Longitudinal changes in cytokine concentrations during therapy

Cytokine concentrations (pg/mL) were longitudinally assessed in three patients over the course of treatment. Seven cytokines of interest are shown. The x-axis represents days from initial diagnosis, and the y-axis indicates cytokine concentrations. Each line represents an individual patient.

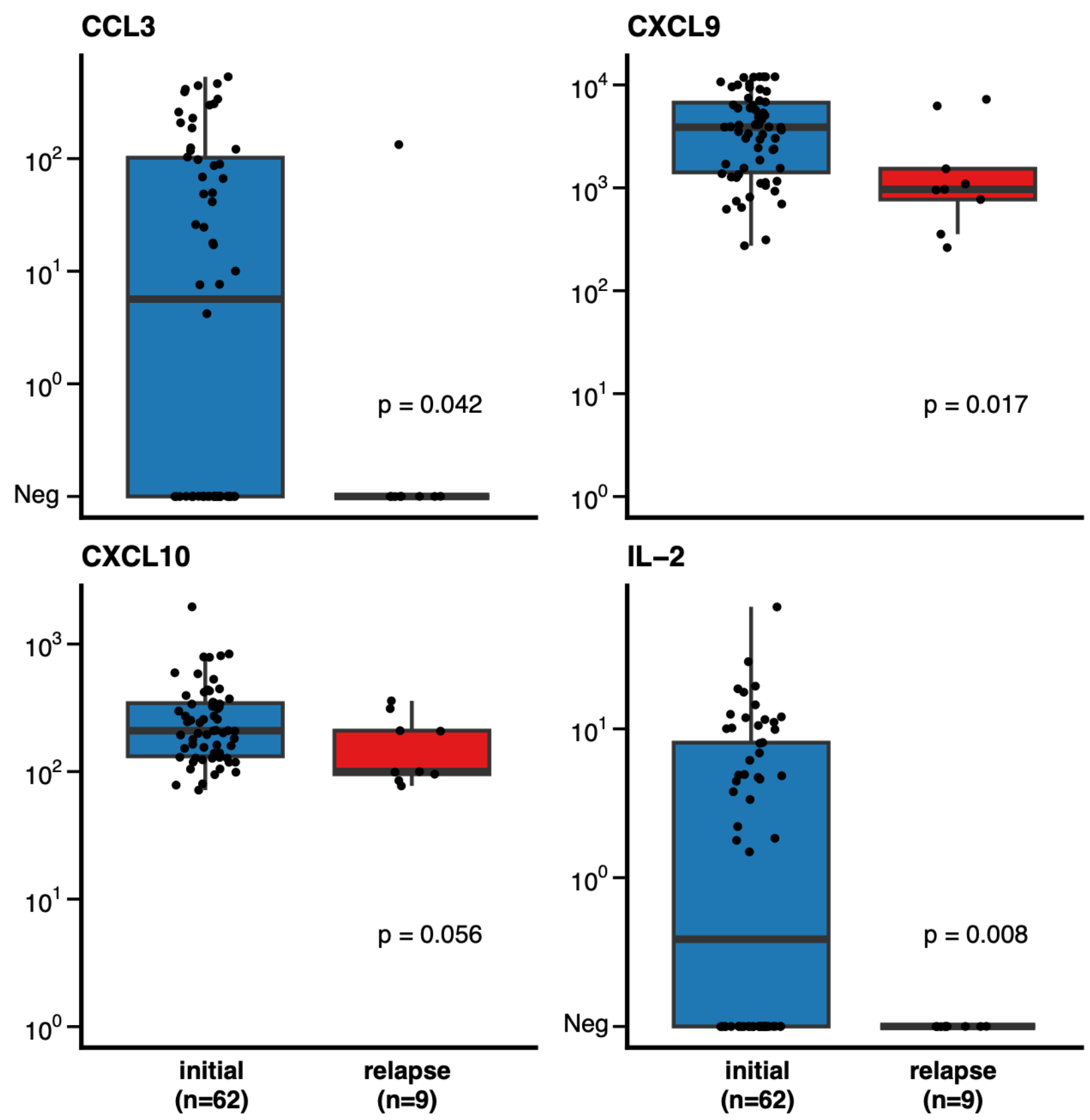

Supplement Figure S9: Cytokine concentrations at initial diagnosis and at relapse

Plasma cytokine concentration measured at initial diagnosis (N=62) and at relapse (N=9). Each dot represents an individual sample, and boxplot show median, interquartile range, minimum and maximum. Cytokine levels are displayed on a logarithmic scale. Only differences reaching statistical significance are shown. Statistical comparisons were performed using Wilcoxon rank-sum test.

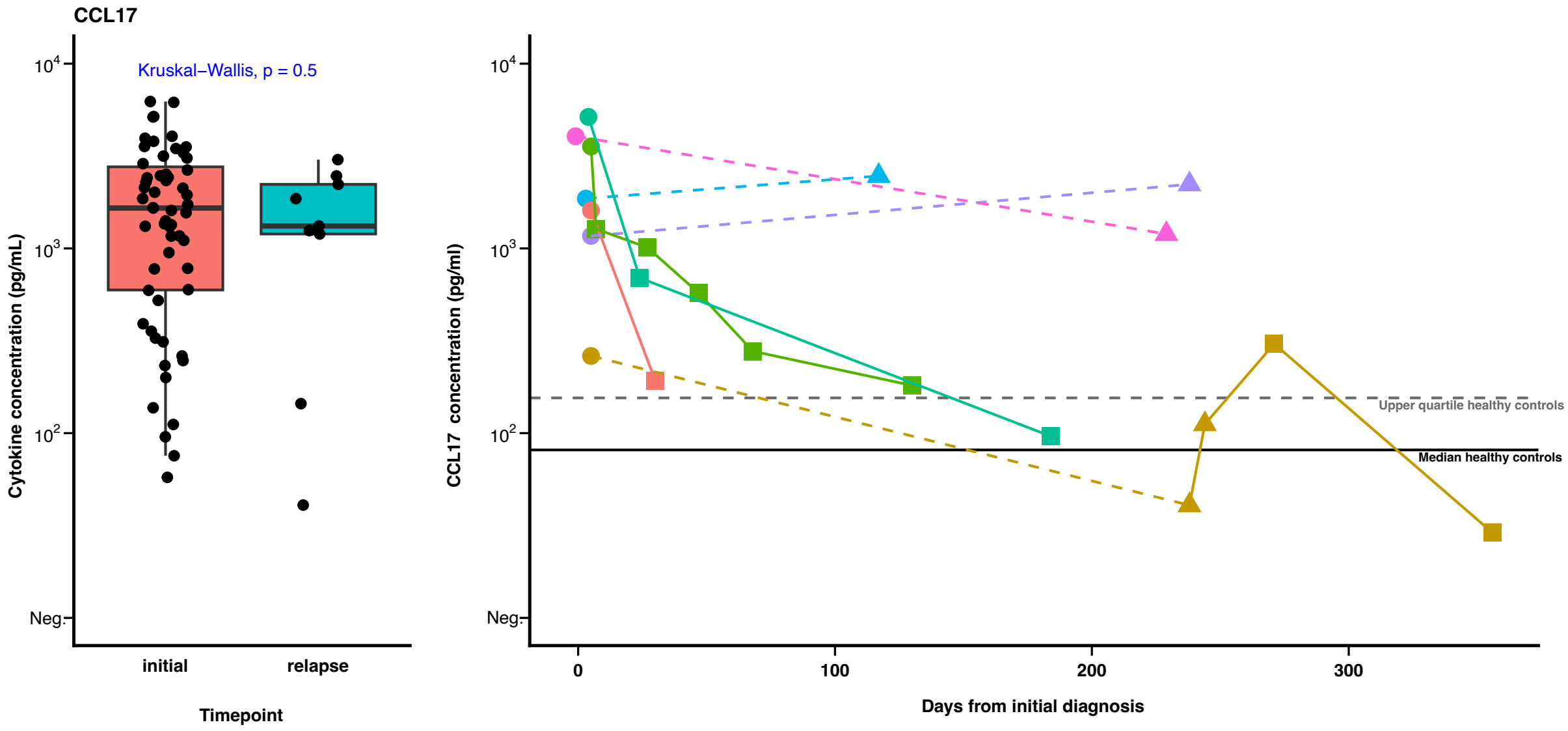

Supplementary Figure S10: CCL17 levels at initial diagnosis, during treatment and at relapse

Comparison of CCL17 levels at initial diagnosis and at relapsed without significant difference (A). Paired samples from four patients comparing initial diagnosis and relapsed with similar CCL17 levels (dotted line). In three patients, CCL17 levels declined during treatment (straight line). In one patient, lower CCL17 level at suspicion of relapse and increased during diagnostics (orange line) (B).

References

1 Griffith, J. W., Sokol, C. L. & Luster, A. D. Chemokines and chemokine receptors: positioning cells for host defense and immunity. *Annu Rev Immunol* **32**, 659-702 (2014). https://doi.org/10.1146/annurev-immunol-032713-120145
2 Groom, J. R. & Luster, A. D. CXCR3 ligands: redundant, collaborative and antagonistic functions. *Immunol Cell Biol* **89**, 207-215 (2011). https://doi.org/10.1038/icb.2010.158
3 Turner, M. D., Nedjai, B., Hurst, T. & Pennington, D. J. Cytokines and chemokines: At the crossroads of cell signalling and inflammatory disease. *Biochim Biophys Acta* **1843**, 2563-2582 (2014). https://doi.org/10.1016/j.bbamcr.2014.05.014
4 Zhu, J. & Paul, W. E. CD4 T cells: fates, functions, and faults. *Blood* **112**, 1557-1569 (2008). https://doi.org/10.1182/blood-2008-05-078154
